# Unavailability of Essential Obstetric Care Services in a Local Government Area of South-West Nigeria

**Published:** 2007-03

**Authors:** Kayode T. Ijadunola, Adesegun O. Fatusi, Ernest O. Orji, Adebanjo B. Adeyemi, Olabimpe O. Owolabi, Ebenezer O. Ojofeitimi, Adekunbi K. Omideyi, Alfred A. Adewuyi

**Affiliations:** ^1^ Department of Community Health, College of Health Sciences, Obafemi Awolowo University, Ile-Ife, Nigeria; ^2^ Osun State Prevention of Maternal Mortality Network, Nigeria; ^3^ Department of Obstetrics and Gynaecology, Obafemi Awolowo University; ^4^ Institute of Public Health, College of Health Sciences, Obafemi Awolowo University; ^5^ Department of Demography and Social Statistics, Faculty of Social Sciences, Obafemi Awolowo University, Ile-Ife

**Keywords:** Essential obstetric care, Health services, Health facilities, Maternal mortality, Nigeria

## Abstract

This paper reports the findings at baseline in a multi-phase project that aimed at reducing maternal mortality in a local government area (LGA) of South-West Nigeria. The objectives were to determine the availability of essential obstetric care (EOC) services in the LGA and to assess the quality of existing services. The first phase of this interventional study, which is the focus of this paper, consisted of a baseline health facility and needs assessment survey using instruments adapted from the United Nations guidelines. Twenty-one of 26 health facilities surveyed were public facilities, and five were privately owned. None of the facilities met the criteria for a basic EOC facility, while only one private facility met the criteria for a comprehensive EOC facility. Three facilities employed a nurse and/or a midwife, while unskilled health attendants manned 46% of the facilities. No health worker in the LGA had ever been trained in lifesaving skills. There was a widespread lack of basic EOC equipment and supplies. The study concluded that there were major deficiencies in the supply side of obstetric care services in the LGA, and EOC was almost non-existent. This result has implications for interventions for the reduction of maternal mortality in the LGA and in Nigeria.

## INTRODUCTION

All pregnant women, even healthy ones, face some risk of complications that can result in death or serious disabilities if not effectively managed. Each year, an estimated 529,000 women die from pregnancy-related causes, with most cases occurring in developing countries of Africa and Asia (1). Yet, the deaths are only a tip of the iceberg; for every death, at least 30 women suffer serious illness or debilitating injuries (2). In many sub-Saharan African countries, many women receive no medical care before, during or after childbirth. Even deliveries in health facilities can be risky due to poor quality of obstetric care. The same pregnancy-related complications that threaten women's survival can also cause death and disability in newborns, while the same health assistance that would save women's lives could also prevent suffering on the part of women who survive and also their newborn babies.

Maternal mortality is very high in Nigeria despite many efforts to reduce its incidence and, generally, to improve maternal health (3). The 1999 Multiple Indicator Cluster Survey (MICS), conducted by the Nigerian Federal Office of Statistics, in collaboration with the United Nations Children's Fund (UNICEF), puts the maternal mortality ratio (MMR) at 704 per 100,000 livebirths, with a wide geographical disparity ranging from 166 per 100,000 livebirths in South-West to 1,549 per 100,000 livebirths in North-East (4). The MICS figure is believed by many reproductive health experts to be an under-estimate. According to the World Health Organization, the maternal mortality ratio of Nigeria was estimated to be 800 per 100,000 livebirths in 2000 (1), and the Federal Ministry of Health recently estimated the ratio at 948 per 100,000 livebirths (5).

One of the critical pathways to reducing maternal mortality is improving the access, use, and quality of skilled services for the treatment of pregnancy and childbirth-related complications (6). One indicator that tries to capture such access is the proportion of pregnant women who deliver with the assistance of a skilled attendant, which is best assessed using population-based surveys. An alternative and highly-recommended approach to monitoring progress in efforts for reduction in maternal mortality is the use of process indicators relating to essential obstetric care (EOC) services, employing a short list of universally-defined ‘signal functions’ to represent EOC (6) and calculating the use of such services by those who need them most. In addition to avoiding the substantial expense involved in generating maternal mortality estimates, this approach of monitoring ‘process indicators’ can alert programme managers, even at the level of the health facility, to areas of the programme that need to be strengthened.

Low level of access to and use of quality reproductive health services play significant roles in high maternal mortality in Nigeria as indicated in the National Reproductive Health Policy (3). Nigeria has, thus, set the goal of reducing maternal mortality by half between 2001 and 2006. This goal is in line with the developmental goals agreed upon by the global community at the millennium summit of the United Nations in 2000 (7). This goal naturally calls for the availability of data, collected periodically and systematically, to determine the progress towards the reduction of maternal mortality. Unfortunately, such good-quality data on maternal mortality are rare in Nigeria. Thus, the current situation in Nigeria calls for the use of cost-effective and simple approaches to urgently establish critical and currently-valid baselines relating to process indicators in efforts for the reduction of maternal mortality. This would form the basis for evaluating the progress in the nearest future and provide a more scientific basis and solid foundation for policy and programme actions both locally at the level of the health facility and centrally at the level of the health ministry. Such approaches would prove invaluable and relevant in Nigeria's efforts for the reduction of maternal mortality.

This study reports the findings at baseline in a multi-phase project aimed at reducing maternal mortality in a local government area (LGA) in South-West Nigeria and would provide data for subsequent evaluation of programmes of intervention. The specific objectives were to determine the availability of EOC services in the LGA and to assess the quality of existing EOC services.

## MATERIALS AND METHODS

### Study location

The study was conducted in Ife South LGA of Osun State, South-West Nigeria. The LGA is essentially rural and has its headquarters at Ifetedo. It has a population of 140,000 based on the 1991 census projections, and 54 public health facilities, and six private health facilities provide orthodox services.

### Study design

This interventional study is being conducted in phases. The first phase, which constituted the focus of this paper, consisted of a baseline health facility and needs assessment survey. Based on the findings of the baseline study, appropriate intervention strategies directed at providing skilled attendance at births (personnel, equipment, and supplies) shall be elaborated in the LGA, and an appropriate policy for programme actions shall be formulated in collaboration with officials of the health departments of the LGA and the State. After an ongoing period of implementation of intervention, a post-intervention survey shall be conducted to evaluate the impact of intervention strategies.

### Study population

All health facilities (public, private, and mission) rendering any form of obstetric care services (antenatal, delivery, or postnatal) in the LGA constituted the sampling frame for the study. This numbered 52 in all, and half of the facilities were selected for inclusion in the study, using the simple random-sampling technique.

### Data-collection instruments

The data-collection instruments consisted of an interviewer-administered, facility-assessment questionnaire and a self-administered skill-assessment questionnaire for the officer-in-charge of each of the selected facilities. The interviewer-administered EOC facility-review questionnaire was adapted from a similar instrument used for the national survey of EOC facilities (8), with the main variables based on the EOC elements specified in the international guidelines for monitoring the availability and use of obstetric services (6). One instrument was applied per facility to either the nursing staff in charge in the case of primary healthcare centres, or the medical director in the case of private health institutions. The questionnaires were administered by trained data collectors who were officials of the National Population Commission and were supervised by members of the Osun State Network for Prevention of Maternal Mortality (NPMM). The data collectors participated in a one-day training prior to the data-collection exercise. The self-administered questionnaire focused on the assessment of the human resources capacity available within the health institution with regard to the performance of EOC activities and was applied to the responsible officer at each facility. Prior to the commencement of data collection, preliminary discussions were held with the officials of the local government health department to familiarize them with the objectives of the study, the relevance, and perceived benefits of the ‘prevention of maternal mortality’ (PMM) programme and to attract political support for subsequent intervention strategies as will be appropriate.

### Data analysis

Data were entered using the EpiInfo software (version 6.3) for personal computers, and entered data were exported to the SPSS software for analysis. Frequency tables and percentages were used for presenting the data. For the purpose of classification, a basic EOC (BEOC) facility is one that performs all the following six listed ‘signal functions’: (a) administers parenteral antibiotics; (b) administers parenteral oxytocic drugs; (c) administers parenteral anti-convulsants; (d) performs manual removal of placenta; (e) performs removal of retained products of conception; and (f) performs assisted vaginal delivery.

A comprehensive EOC (CEOC) facility, in addition to carrying out all the six listed functions for BEOC facilities, will also (a) perform surgery (caesarian section) and (b) perform safe blood transfusion

## RESULTS

The primary healthcare centres contributed about 69% of the health facilities surveyed, and all these were being operated by the Ife South LGA health department. The Osun State health ministry operated two of the health facilities, and both were comprehensive health centres. Private practitioners were running five healthcare facilities (4 maternity centres and 1 comprehensive health centre) (results not shown).

The EOC services that were most routinely performed by the health facilities (Table 1) included: administration of parenteral oxytocics (by about 81% of facilities), manual removal of placenta (34.6% of facilities), and administration of parenteral antibiotics (30.8%). On the other hand, the services that were most commonly performed in the three months preceding the survey included: administration of parenteral oxytocics (65.4% of facilities), administration of parenteral antibiotics (19.2% of facilities), and assisted vaginal deliveries (15.4% of facilities). The services that were least routinely performed were: blood transfusion (reported by only three facilities) and caesarean section, reported by only one private centre. None of the facilities concerned had carried out any blood transfusion or caesarean section in the three months preceding the survey. Among the private facilities, only one met the criteria for CEOC, while none met the criteria for BEOC. On the other hand, none of the 21 public-sector facilities met the criteria for either BEOC or CEOC. On the whole, none of the facilities surveyed was a BEOC facility, while one (1.9%) was a CEOC facility.

**Table 1.** Number of health facilities (n=26) that performed EOC services routinely and within the three months preceding the survey, Ife South LGA, Nigeria

Service checklist	Facilities where services are routinely performed		Facilities where services were performed in the 3 months preceding the survey	
	No.	%	No.	%
Parenteral antibiotics	8	30.8	5	19.2
Parenteral oxytocics	21	80.8	17	65.4
Parenteral sedatives	7	26.9	3	11.5
Manual removal of placenta	9	34.6	3	11.5
Removal of retained products of conception	4	15.4	1	3.8
Assisted vaginal delivery	2	7.7	4	15.4
Blood transfusion	3	11.5	0	0
Caesarean section	1	3.8	1	3.8

EOC=Essential obstetric care;

LGA=Local Government Area

In 81% of the health facilities, health workers ran morning, afternoon, and night shifts, and in five (19%) facilities, workers ran either morning only or morning and afternoon only shifts (results not shown). There was a dearth of qualified personnel in the health facilities at the Ife South LGA, as only three facilities had at least a nurse and/or a midwife in their employment, and none had the minimum of four midwives required to ensure 24-hour coverage by skilled attendants (Table 2). One privately-owned facility employed a doctor, while semi-skilled community health extension workers (CHEWs) manned the bulk of the health centres. Even then, about 46% of the facilities employed no CHEWs and so were being manned by unskilled health assistants. No health worker of any cadre in any of the health facilities had been trained in lifesaving skills in the LGA. The main reasons given for the non-availability of the various cadres of staff included non-employment by the authorities (in over 90% of cases) and recent transfers of staff to other locations.

**Table 2.** Availability of skilled personnel in the health facilities of Ife South LGA, Nigeria (number of facilities reporting=24)

Trained personnel available	No. of facilities employing skilled personnel	Percentage
CHEWs		
None	11	45.8
1	4	16.7
2	7	29.2
4	1	4.2
6	1	4.2
CHOs		
None	19	79.2
1	4	16.7
2	1	4.2
Midwives		
None	21	87.5
1	2	8.3
2	1	4.2
Nurses		
None	21	87.5
1	1	4.2
2	1	4.2
3	1	4.2
Doctor	1	3.8
Obstetrician	0	0.0
Anaesthetist	0	0.0

CHEWs=Community Health Extension Workers;

CHOs=Community Health Officers;

LGA=Local Government Area

In many facilities in Ife South LGA where the drugs needed to provide essential obstetric care services were available, the drugs were provided in the labour wards/maternity units. Parenteral oxytocics were available in only half of the facilities, while parenteral anti-convulsants for the management of hypertensive diseases of pregnancy were available in 11.5% of the facilities. Of the parenteral antibiotics, metronidazole was the most commonly provided, being available in 61.5% of the health facilities, while other antibiotics (e.g. penicillins) were available in less than half of the facilities. Further assessment of the availability of equipment and supplies necessary for EOC services (Table 3) showed that intravenous infusion fluids were not available in 20 (77%) health facilities, and there were no latex gloves in more than two-thirds of the facilities. In 25 (96%) facilities, partographs were neither available nor being used for monitoring the progress of labour, while nearly two-thirds of the facilities had no sterilizers. Half of the facilities did not have sterile syringes and needles, while 69% had no vaginal speculums. More than a quarter (27%) of the facilities had no sphygmomanometers to monitor the blood pressure of obstetric clients under their care.

**Table 3.** Availability of equipment and supplies for EOC services in health facilities (n=26) of Ife South LGA, Nigeria

Labour room supplies/equipment	Facilities where item is available		Facilities where item is not available	
	No.	%	No.	%
IV infusion set	6	23.0	20	77.0
IV fluid	6	23.0	20	77.0
Vacuum extractor	7	27.0	19	73.0
Latex glove	8	31.0	18	69.0
Partograph	1	4.0	25	96.0
Manual vacuum aspirator	2	8.0	24	92.0
Ovum forceps	8	31.0	18	69.0
Suture	10	38.0	16	62.0
Syringes and needles	13	50.0	13	50.0
Sterilizer	9	35.0	17	65.0
Scissors	18	69.0	8	31.0
Curette	2	8.0	24	92.0
Sphygmomanometer	19	73.0	7	27.0
Vaginal speculum	8	31.0	18	69.0

EOC=Essential obstetric care;

IV=Intravenous;

LGA=Local Government Area

In the year preceding the survey, no deliveries were recorded in five health facilities in the LGA. Eight (31%) facilities recorded 1–10 birth(s), one facility (3.8%) recorded 41–50 births, and two (7.7%) facilities recorded 71–90 births. Assisted vaginal deliveries were performed in only two (7.7%) facilities during the same period; one facility carried out two assisted vaginal deliveries, while another had four cases. Furthermore, only three (11.5%) facilities had performed a caesarian section in the 12 months preceding the survey, and all these were privately owned. In all, these facilities had performed 2–4 caesarian sections each during the time period. Also, only five (19.2%) health facilities had treated women for complications of pregnancy in the 12 months preceding the survey, the remaining 21 (80.8%) performed no such functions over the time period.

Table 4 shows that only one respondent (the Medical Officer of Health in the LGA) of the 26 heads of facilities interviewed had undergone any competency-based training on the job for the improvement of obs-tetrics-related skills. Only half of the respondents had conducted deliveries in the month preceding the survey, while about 31% had not conducted deliveries in the six months preceding the survey. Consistently, less than one-fifth of the respondents had ever cared for patients with postpartum haemorrhage, obstructed labour, puerperal sepsis, eclampsia, complications of unsafe abortion, or retained placenta. Similarly, only 11.5% of the respondents had ever used partograph to monitor the progress of labour.

**Table 4.** Performance of selected EOC services by interviewed health workers in Ife South LGA, Nigeria (n=26)

Activity	No.	Percentage
Proportion of health workers who have undergone updated courses on the job	1	3.8
Proportion of health workers who took deliveries in the month preceding the survey	13	50.0
Proportion of respondents who have ever provided care for postpartum haemorrhage	5	19.2
Proportion of respondents who have ever provided care for obstructed labour	3	11.5
Proportion of health workers who have ever provided care for puerperal sepsis	4	15.4
Proportion of health workers who have ever provided care for eclampsia	1	3.8
Proportion of health workers who have ever provided care for complication of unsafe abortion	2	7.7
Proportion of health workers who have ever provided care for retained placenta	4	15.4
Proportion of health workers who had ever used the partograph to monitor labour	3	11.5

EOC=Essential obstetric care;

LGA=Local Government Area

## DISCUSSION

The findings of this study showed that EOC services are still not readily available in Nigeria despite the provisions of the National Reproductive Health Policy to reduce the number of deaths due to pregnancy and child birth by half between 2001 and 2006 (3). This was despite the fact that the study was conducted in the southwestern zone of the country, a zone that recorded the least maternal mortality estimate of 166 per 100,000 livebirths in the 1999 MICS (4).

Skilled attendance at birth and the provision of quality EOC are recognized as crucial correlates of birth outcomes worldwide (9, 10), as most obstetric complications are unpredictable and tend to occur during labour and immediately after delivery. It requires a skilled professional to swiftly recognize life-threatening complications and to intervene in time to save both mother and baby (11, 12). It has been estimated that, if 15% of all births are attended by doctors and 85% of them are attended by midwives, the rate of maternal mortality will be adequately reduced. This ratio is most effective in situations where midwives attend normal deliveries and are able to effectively refer 15% of deliveries that result in complications to physicians (11). This study clearly revealed a dearth of trained skilled personnel in the health facilities due to non-employment by the authorities in over 90% of cases. This is a common picture in several health facilities (both primary and secondary) in all geopolitical zones of Nigeria (8). While midwives are continuously being trained in the country and several of them are without employment, the local government authorities employ a preponderance of health attendants with no skills at all, and few CHEWs with limited skills to work in the Primary Health Centres (PHCs), which are the nearest facilities to the people. This also, sometimes, happens in state-owned general/district hospitals, which are supposed to be referral centres. The explanations usually given by the authorities when confronted with evidence of this unwholesome practice include “an embargo on the employment of skilled personnel in the Local Government Areas by the State governments” and “the high costs of hiring a midwife compared to health attendants and CHEWs”.

It was, therefore, not surprising that none of the health facilities surveyed qualified to be a BEOC facility, while only one private facility qualified for the CEOC status. The findings of the study contrasted sharply with the government policy that prescribes the establishment of four BEOC and one CEOC facilities per 500,000 population and the employment of at least four midwives per BEOC facility. It then only confirms that most facilities in Nigeria cannot effectively respond to obstetric emergencies and, thereby, avert maternal deaths as had been earlier reported (8). In the study communities, pregnant women needing EOC may even choose not to visit the health facility, even when they are near, if they know that such facilities lack qualified personnel with obstetric skills (13). Where potential patients have access to more than one facility or service provider, their perception of the quality of care offered at these facilities often takes precedence over concerns about distance. Despite the dearth of skilled personnel, significant proportions of the facilities surveyed carried out such specialized procedures as administration of parenteral oxytocics, manual removal of placenta, administration of parenteral antibiotics, and assisted vaginal deliveries. This only translates to the fact that the auxillary staff who man many facilities, none of whom had been exposed to livesaving skills training, perform these procedures. This is a dangerous trend, and one that can actually increase the burden of maternal morbidity and mortality that the health facilities should ordinarily be addressing.

A lack of equipment and supplies observed in this survey plagues health facilities in most regions of the developing world, and such shortages occur at all levels of the health system in Nigeria (14). There is little question that this situation is due, in part, to the challenge of limited resources. However, it is often compounded by ineffective organization and poor management of the available resources. The inadequate supplies of essential drugs, such as antibiotics, oxytocics, and anti-convulsants, observed in this survey, are other avoidable factors that contribute to phase 3 delays associated with maternal mortality. In 2003, it was estimated that one in every 100 Nigerian women (about 54,000 women) died tragically as a result of pregnancy, delivery, or the aftermath, because the country failed to give them quality, socially-acceptable, and accessible maternal health services (15). The National HIV/AIDS and Reproductive Health Survey (NARHS) of 2003 revealed that the use of maternal healthcare services was quite low. Although 62% of women in the NARHS reported attending an antenatal care clinic at least once during pregnancy, only 34% had skilled attendants at birth and only 41% received postnatal care. The use of maternal health services was higher in urban than in rural areas. Compared to 1999, there was a decline in the use of maternal health services in 2003. If this trend continues, Nigeria's plan to significantly reduce maternal mortality by 2015 will be in serious jeopardy.

This survey identified major deficiencies in the supply side of obstetric care services being offered in the Ife South LGA of Osun State of Nigeria, with EOC being almost non-existent and quality of services being quite poor. This trend, common among the primary and secondary facilities all over the country, is an indication of the dreadful state of health services in Nigeria. It is advocated that the simple and effective strategy of ensuring the presence of skilled attendants at every delivery be pursued by all levels of service-delivery in the country. Meanwhile, based on the population of different LGAs and States, certain health facilities should be upgraded to the status of BEOCs and CEOCs that will serve as referral facilities for obstetric complications and emergencies. This will require the deployment of minimum requirements of skilled staff with their supportive supervision, the provision of necessary equipment and supplies, and a total refurbishment of the infrastructure and support services of such designated facilities.

## ACKNOWLEDGEMENTS

This study was funded through a grant from the Nigerian Prevention of Maternal Mortality Network to the Osun State Network for the Prevention of Maternal Mortalty.
